# Association between solar radiation and mood disorders among Gulf Coast residents

**DOI:** 10.1038/s41370-024-00691-w

**Published:** 2024-06-03

**Authors:** Xinlei Deng, Lenore J. Launer, Kaitlyn G. Lawrence, Emily J. Werder, Ian D. Buller, William Braxton Jackson, Dale P. Sandler

**Affiliations:** 1 Epidemiology Branch, National Institute of Environmental Health Sciences, Durham, NC USA; 2 Laboratory of Epidemiology and Population Sciences, National Institute on Aging, Bethesda, MD USA; 3 Social & Scientific Systems, Inc., a DLH Holdings Company, Silver Spring, MD USA; 4 Social & Scientific Systems, Inc., a DLH Holdings Company, Durham, NC USA

**Keywords:** Meteorological conditions, Solar radiation, Psychological distress, Depression

## Abstract

**Background:**

Climate factors such as solar radiation could contribute to mood disorders, but evidence of associations between exposure to solar radiation and mood disorders is mixed and varies by region.

**Objective:**

To evaluate the association of solar radiation with depression and distress among residents living in U.S. Gulf states.

**Methods:**

We enrolled home-visit participants in the Gulf Long-Term Follow-up Study who completed validated screening questionnaires for depression (Patient Health Questionnaire-9, *N* = 10,217) and distress (Kessler Psychological Distress Questionnaire, *N* = 8,765) for the previous 2 weeks. Solar radiation estimates from the Daymet database (1-km grid) were linked to residential addresses. Average solar radiation exposures in the seven (SRAD7), 14 (SRAD14), and 30 days (SRAD30) before the home visit were calculated and categorized into quartiles (Q1-Q4). We used generalized linear mixed models to estimate prevalence ratios (PR) and 95% confidence intervals (CI) for associations between solar radiation and depression/distress.

**Results:**

Higher levels of SRAD7 were non-monotonically inversely associated with depression [PR_Vs.Q1_ (95%CI): Q2 = 0.81 (0.68, 0.97), Q3 = 0.80 (0.65, 0.99), Q4 = 0.88 (0.69, 1.15)] and distress [PR_Vs.Q1_ (95%CI): Q2 = 0.76 (0.58, 0.99), Q3 = 0.77 (0.57, 1.06), Q4 = 0.84 (0.58, 1.22)]. Elevated SRAD14 and SRAD30 appeared to be associated with decreasing PRs of distress. For example, for SRAD14, PRs were 0.86 (0.63–1.19), 0.80 (0.55–1.18), and 0.75 (0.48–1.17) for Q2-4 versus Q1. Associations with SRAD7 varied somewhat, though not significantly, by season with increasing PRs of distress in spring and summer and decreasing PRs of depression and distress in fall.

**Impact Statement:**

Previous research suffered from exposure misclassification, which impacts the validity of their conclusions. By leveraging high-resolution datasets and Gulf Long-term Follow-up Cohort, our findings support an association between increased solar radiation and fewer symptoms of mood disorders.

## Introduction

Psychological distress and depression are two types of mood disorders characterizing disturbances in emotions. Psychological distress refers to non-specific symptoms of stress, anxiety, and depression. In the National Health Interview Survey, the prevalence of psychological distress in the past 30 days increased from 16.1% in 1999 to 22.6% in 2018 [1]. Studies have shown that psychological distress is closely associated with cardiovascular and respiratory diseases, cancers, and changes in immune responses [2–6]. While distress may be short-term or situational, depression is typically more severe and long-term and is different from usual mood fluctuations. A systematic review reported that the prevalence of symptoms of depression was about 8.8% [7]. Depression can also negatively impact health including cardiovascular and respiratory diseases and cancers, and every domain of daily life including work, school, and family [8].

Scientific evidence suggests that some mental disorders could be attributed to climate factors [9]. However, previous studies evaluating links between solar radiation and mood disorders are mixed and results vary by geographic region [10–17]. Some studies found beneficial effects of solar radiation on mood disorders including depression [10, 13]. For instance, Kim et al. [13] reported that a shorter duration of sunshine was related to an increased risk of depression [13]. On the other hand, Henríquez-Sánchez et al. [17] found that participants from Southern Spain exposed to higher levels of solar radiation were more likely to report depressive symptoms such as feelings of worthlessness or guilt [17], although annual solar radiation prior to symptom assessment was not associated with the total number of depressive symptoms [16].

Different findings across studies may reflect differences in methods used to evaluate the effects of solar radiation on mood disorders. First, studies differed in the number of solar radiation days used (from one week to one year) [10, 13, 15, 16]. In addition, previous findings were based on case-control design or suffered from a limited sample size [15, 16]. Further, many studies were conducted in high-latitude areas [10, 13, 15]. The impact of solar radiation on mood disorders among participants with a relatively high background in solar radiation is understudied. More importantly, some studies used meteorological data from the nearest weather monitoring station or at a relatively low spatial resolution. In Son’s study, the distance between the stations where sunlight was measured ranged from 13 to 69 km [15]. Komulainen et al. used meteorological measurements at the zip code level [16]. In these studies, exposure misclassification and the ‘ecologic fallacy’ could impact the validity of their conclusions.

We aimed to add to and improve on this literature by evaluating the association between solar radiation and symptoms related to mood disorders among residents living in the five Gulf states who participated in the Gulf Long-Term Follow-up Study using a more spatially resolved measure of solar radiation (1 km x 1 km).

## Methods

### Study design and participants

The Gulf Long-term Follow-up (GuLF) Study is a prospective cohort study designed to examine human health effects following the 2010 *Deepwater Horizon* oil spill in the Gulf of Mexico. The GuLF Study includes 32,608 adults ≥ 21 years of age who participated in oil spill response and cleanup work (N = 24,937) and those who trained for potential work but were not hired (*N* = 7671) [18]. Participants from across the U.S., but largely from the Gulf region, were enrolled between March 2011 and March 2013 and completed a 30- to 60-minute computer-assisted telephone enrollment interview. The interview collected information on oil spill response and cleanup activities, and demographic, socioeconomic, occupational, lifestyle, and health information [18]. Study questionnaires can be found at www.niehs.nih.gov/gulfstudy. Participants from eastern Texas, Louisiana, Mississippi, Alabama, and Florida (Gulf Coast region) were invited to have a home visit (*N* = 26,828). A total of 11,119 completed the home visit (May 2011-May 2013) which included collection of biological samples, functional measures (e.g., blood pressure, lung function), and anthropometrics, and examiner-administered questionnaire data, including mental health screeners. Home visit participants provided written informed consent. The study was approved by the Institutional Review Board of the National Institutes of Health.

### Outcome ascertainment

For this analysis, we were interested in two primary mental health symptoms: depression and psychological distress. We used the original questionnaires of Patient Health Questionnaire-9 (PHQ-9) for depression and Kessler Quick Inventory of Distress (K6) for psychological distress as they have strong validity and reliability (questionnaire is available at https://epishare.niehs.nih.gov/studies/GuLF/).

Specifically, the PHQ-9 was used to estimate current depression at the time of home visit by assessing symptoms that occurred over the past two weeks. PHQ-9 is the 9-item depression severity measure adapted from the full PHQ. Each item of PHQ-9 is scored from 0 (not at all) to 3 (nearly every day) and the total score can range from zero to 27. Studies showed that compared to an independent structured mental health professional interview, a PHQ-9 score ≥10 yielded a sensitivity of 88% and a specificity of 88% for major depression [19]. We used this threshold to categorize the participants as “depressed” and “not depressed”. There were 10,217 participants who completed PHQ-9 and had complete data for all other covariates.

We used the K6 to evaluate current psychological distress at the time of home visit [20]. K6 has been widely used and validated to assess non-specific psychological distress symptoms such as feelings of nervousness, hopelessness, and worthlessness. Except for “don’t know” and “refused”, the responses range from “none of the time” to “all of the time”. The six items in K6 are summed to a score ranging from zero to 24. Previous studies have demonstrated that K6 has excellent internal consistency and reliability. Prior validation studies of the K6 against clinical structured diagnosis reported an accuracy of 0.92 at a cut‐point ≥ 13 [20, 21]. Again, we dichotomized the scores of psychological distress based on this threshold. The K6 was added to the home visit sometime after the home visits had started. Thus, there were 8,765 participants who completed the K6 and had complete information on all relevant covariates.

### Exposure characterization

We obtained solar radiation exposure estimates (Watt/m^2^) from the newly updated Daily Surface Weather Data (Daymet Version 4 R1) database [22]. Daymet is a high-quality research product of the Environmental Sciences Division at Oak Ridge National Laboratory and is supported by the National Aeronautics and Space Administration (NASA) through the Earth Science Data and Information System (ESDIS) and the Terrestrial Ecology Program that combines measured data with models to achieve 1 km x 1 km grid estimates of climate factors [22]. The estimates from Daymet can have a correlation with observations up to a high R^2^ of 98.9% [23]. Daymet provides information on daily minimum temperature, maximum temperature, precipitation, shortwave radiation, vapor pressure, snow water equivalent, and day length from 1980 to 2021. The gridded datasets were spatially linked to participants’ geocoded home visit residential addresses. We calculated the average solar radiation (SRAD) exposure over the seven (SRAD7), 14 (SRAD14), and 30 (SRAD30) calendar days before each participant’s home visit. SRAD7, SRAD14, and SRAD30 were categorized into quartiles (Q1, Q2, Q3, and Q4) based on the empirical distribution among the analytic sample. Temperature and humidity were also obtained from the Daymet database.

### Statistical analysis

We analyzed associations between mental health status and residential solar radiation exposures in the past seven (SRAD7), 14 (SRAD14), and 30 days (SRAD30) prior to the home visit. We generated maps showing the average values of the SRAD7, SRAD14, and SRAD30 that participants in each county/Parish were exposed to. We used crude and adjusted generalized linear mixed models to estimate prevalence ratios (PR) and 95% confidence intervals (CI) for associations between solar radiation and depression and distress. We developed three models: 1) crude models; 2) models adjusted for age groups (20-40, 40-60, and >60), self-reported race (White, Black, and other), ethnicity (Hispanic, non-Hispanic), self-reported sex (female, male), education (less than high school, high school, some college, and college or greater), employment status at the time of enrollment (yes, no), ever alcohol consumption (yes, no), ever cigarette smoking (yes, no), *Deepwater Horizon* oil spill cleanup worker status (yes, no), season (spring, summer, fall, and winter), residence in a county or Parish abutting the Gulf of Mexico (“proximity”; yes, no), and state of residence (as a random effect); and 3) models additionally adjusting for temperature and relative humidity with corresponding time lags [10, 24]. We did not adjust for income because of a high proportion of missing values (*N* = 787, 7.1%). Instead, we carried out a sensitivity analysis and found similar results in models with and without adjustment for income where all other covariates were controlled (Supplementary Table A1).

We used a directed acyclic graph to show the presumed relationships among potential confounders (Supplementary Fig. A1). In addition, we conducted stratified analyses to evaluate effect modification by season, age group, and sex [25]. For the age we stratified the participants at age 50, the approximate median age of the cohort, which gave us reasonable sample size in each age stratum. We also conducted a sensitivity analysis among participants who had complete data for both outcomes and all other covariates (Supplementary Fig. A2). Statistical significance was defined as *p* value < 0.05. All analyses were conducted in R version 4.2.1.

## Results

**Fig. 1:**
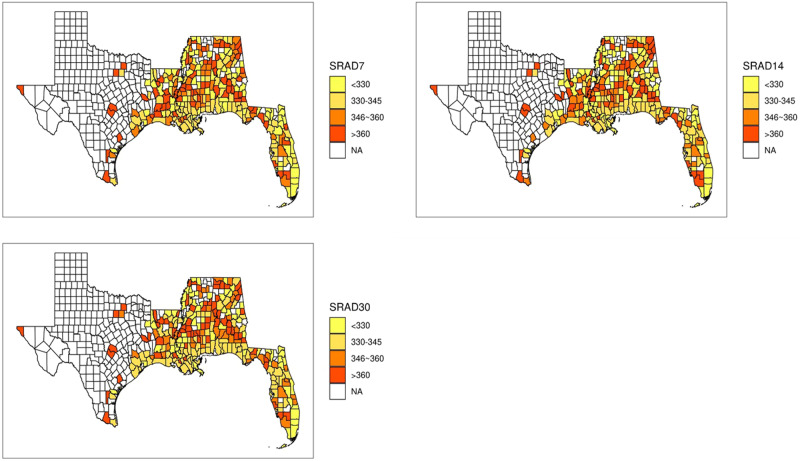
Spatial distribution of solar radiation (Watt/m^2^) at the county level among Gulf coast participants during 2011–2013. SRAD7: average solar radiation over the seven calendar days before home visits; SRAD14: average solar radiation over the 14 calendar days before home visits; SRAD30: average solar radiation over the 30 calendar days before home visit.

Among a total of 11,119 home-visit participants, about 78% of the participants were male and with an average age of 43.8 years. More than half of the sample was White (54.8%) and non-Hispanic (93.9%). Table 1 shows the distribution of demographic characteristics by depression and distress among the home-visit participants. Among all participants, 10,217 participants with complete covariates had PHQ-9 and 8,765 participants had K6. Symptoms assessed in the two scales are somewhat overlapping. The correlation between the PHQ-9 and K6 was 0.79. Among 8,464 participants with responses for both scales, 740 were positive for depression alone, 100 were positive for distress alone, and 529 were both distressed and depressed. Generally, participants who were not depressed or distressed were more likely to have a higher level of education, be employed, have a higher income, and smoke less. Figure 1 presents the spatial distribution of SRAD7, SRAD14, and SRAD30 at the county/Parish level among Gulf Coast participants during 2011-2013. The spatial patterns of SRAD7, SRAD14, and SRAD30 were similar and showed considerable exposure variations across the study area.

**Table 1:** Demographic characteristics by depression and distress among GuLF coast residents.

Group		Whole population (*N* = 11,119)	Depression (*N* = 10,217)			Distress (*N* = 8765)		
			No	Yes	*P* value	No	Yes	*P* value^*^
Age (years old)	20-40	4574 (41.1%)	3558 (41.5%)	651 (39.5%)	<0.001	3370 (41.7%)	282 (41.4%)	<0.001
	41-60	5413 (48.7%)	4079 (47.6%)	907 (55.0%)		3889 (48.1%)	365 (53.5%)	
	>60	1132 (10.2%)	930 (10.9%)	92 (5.6%)		824 (10.2%)	35 (5.1%)	
Gender	Male	8696 (78.2%)	6765 (79.0%)	1220 (74.0%)	<0.001	6337 (78.4%)	491 (72.0%)	<0.001
	Female	2423 (21.8%)	1802 (21.0%)	430 (26.0%)		1746 (21.6%)	191 (28.0%)	
Race	White	6065 (54.8%)	4680 (54.6%)	880 (53.3%)	0.03	4321 (53.5%)	374 (54.8%)	0.73
	Black	3864 (34.9%)	2995 (35.0%)	625 (37.9%)		2919 (36.1%)	242 (35.5%)	
	Other	1144 (10.3%)	892 (10.4%)	145 (8.8%)		843 (10.4%)	66 (9.7%)	
Ethnicity	Hispanic	672 (6.1%)	538 (6.3%)	66 (4.0%)	<0.001	533 (6.6%)	31 (4.6%)	0.04
	Non-Hispanic	10,417 (93.9%)	8029 (93.7%)	1584 (96.0%)		7550 (93.4%)	651 (95.5%)	
Education	< high school	2361 (21.3%)	1676 (19.6%)	473 (28.7%)	<0.001	1668 (20.6%)	219 (32.1%)	<0.001
	High school	3771 (34.0%)	2878 (33.6%)	606 (36.7%)		2748 (34.0%)	247 (36.2%)	
	Some college	3327 (30.0%)	2613 (30.5%)	464 (28.1%)		2432 (30.1%)	183 (26.8%)	
	≥ College	1626 (14.7%)	1400 (16.3%)	107 (6.5%)		1235 (15.3%)	33 (4.8%)	
Employment	Yes	5926 (53.5%)	4841 (56.5%)	649 (39.3%)	<0.001	4462 (55.2%)	242 (35.5%)	<0.001
	No	5152 (46.5%)	3726 (43.5%)	1001 (60.7%)		3621 (44.8%)	440 (64.5%)	
Oil spill	Yes	8917 (80.2%)	6820 (79.6%)	1370 (83.0%)	0.002	6502 (80.4%)	557 (81.7%)	0.47
work status	No	2202 (19.8%)	1747 (20.4%)	280 (17.0%)		1581 (19.6%)	125 (18.3%)	
Alcohol	Yes	10,251 (92.5%)	7944 (92.7%)	1519 (92.1%)	0.37	7470 (92.4%)	619 (90.8%)	0.14
	No	827 (7.5%)	623 (7.3%)	131 (7.9%)		613 (7.6%)	63 (9.2%)	
Smoking	Yes	6370 (57.5%)	4739 (55.3%)	1,096 (66.4%)	<0.001	4490 (55.6%)	475 (69.7%)	<0.001
	No	4699 (42.5%)	3828 (44.7%)	554 (33.6%)		3593 (44.5%)	207 (30.4%)	
Annual Income	≤$20,000	4142 (40.1%)	2969 (38.3%)	752 (49.8%)	<0.001	2870 (39.2%)	331 (53.7%)	<0.001
	$20,001-$50,000	3443 (33.3%)	2542 (32.8%)	518 (34.3%)		2439 (33.3%)	205 (33.2%)	
	>$50,000	2747 (26.6%)	2238 (28.9%)	240 (15.9%)		2008 (27.4%)	81 (13.1%)	
Season	Spring	2532 (22.8%)	1958 (22.9%)	377 (22.9%)	0.83	2158 (26.7%)	199 (29.2%)	0.30
	Summer	2740 (24.6%)	2123 (24.8%)	393 (23.8%)		2011 (24.9%)	163 (23.9%)	
	Fall	2992 (26.9%)	2306 (26.9%)	458 (27.8%)		2034 (25.2%)	154 (22.6%)	
	Winter	2855 (25.7%)	2180 (25.5%)	422 (25.6%)		1880 (23.3%)	166 (24.3%)	
Proximity	Yes	8101 (72.9%)	6166 (72.0%)	1272 (77.1%)	<0.001	5787 (71.6%)	538 (78.9%)	<0.001
	No	3018 (27.1%)	1799 (28.0%)	226 (22.9%)		1720 (28.4%)	144 (21.1%)	

^*^Chi-square tests.

**Fig. 2:**
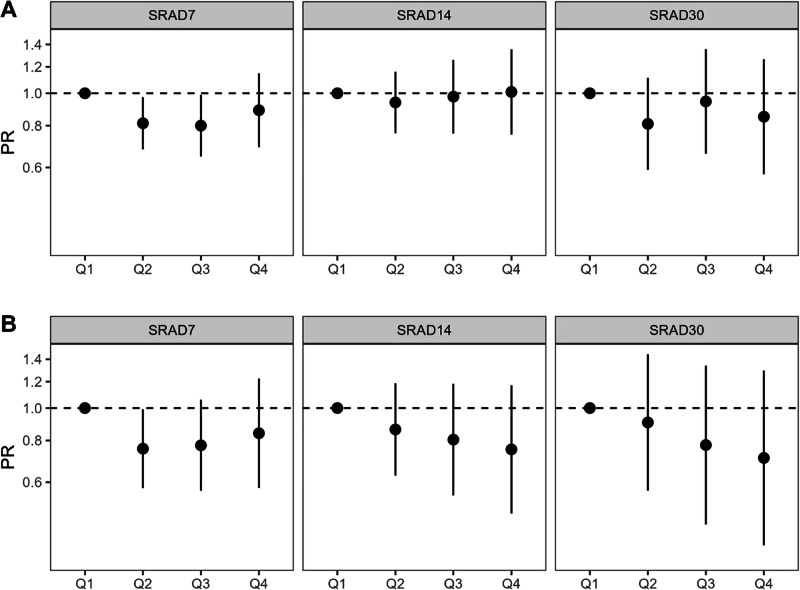
Association of depression and distress with quartiles of average solar radiation in the past seven, 14 and 30 days among GuLF study participants. **A**: depression (*N* = 10,217); **B**: distress (*N* = 8,765).

In the fully adjusted models also controlling for the effects of temperature and relative humidity, higher levels of SRAD7 were non-monotonically inversely associated with depression (PR_Vs.Q1_ (95%CI): Q2 = 0.81 (0.68, 0.97), Q3 = 0.80 (0.65, 0.99), Q4 = 0.89 (0.69, 1.15)) and distress (PR_Vs.Q1_ (95%CI): Q2 = 0.76 (0.58, 0.99), Q3 = 0.77 (0.57, 1.06), Q4 = 0.84 (0.58, 1.22)) (Fig. 2). For SRAD14, PRs were 0.86 (0.63-1.19), 0.80 (0.55-1.18), and 0.75 (0.48-1.17) for Q2-4 versus Q1, and for SRAD30, PRs were 0.91 (0.57-1.45), 0.78 (0.45-1.34), and 0.71 (0.39-1.29) for Q2-4 versus Q1 (Fig. 2). Although these trends are not statistically significant (P for trend in the Supplementary Fig. A3), we did observe potential decreasing trends of SRAD14 and SRAD30 for distress. Results were similar in analyses limited to participants with data for both outcomes (Supplementary Fig. A2). Complete results were presented in Supplementary Table A2.

**Fig. 3:**
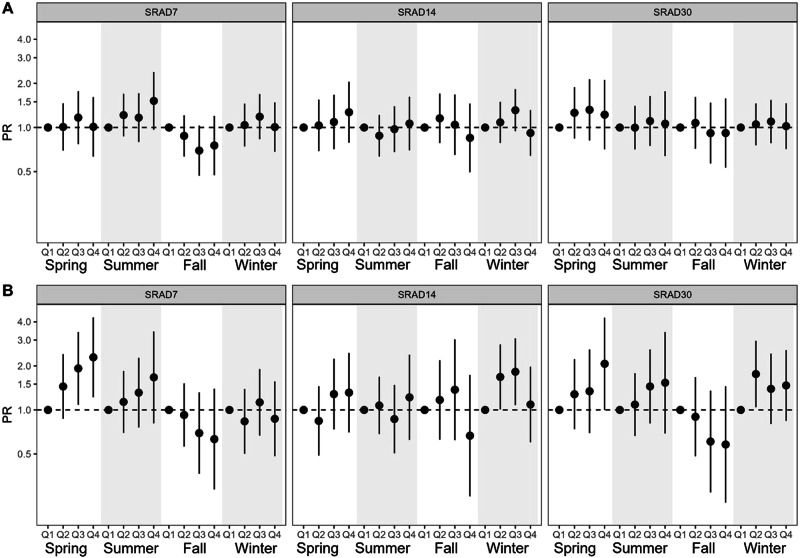
Association of depression and distress with quartiles of average solar radiation in the past seven, 14, and 30 days among GuLF study participants stratified by season. Panel A: depression (*N* = 10,217); Panel B: distress (*N* = 8765).

Seasonal variation was more apparent for distress than depression. For distress, the associations in Spring and Summer were qualitatively different from those in Fall (Fig. 3). For SRAD7 and SRAD30, there was an apparent trend of increased PRs in Spring and Summer (PR_Vs.Q1_ range in Spring = 1.28–2.29; PR_Vs.Q1_ range in Summer = 1.09–1.67) with increasing solar radiation, whereas in Fall, both depression and distress were non-significantly decreased with increasing SRAD7 (Depression: PR_Vs.Q1_ range = 0.70–0.88; Distress: PR_Vs.Q1_ range = 0.63–0.92).

Results from analyses stratified by age group and sex are presented in the supplemental materials. While not statistically significant, PRs for distress were reduced at higher levels of SRAD14 and SRAD30 among those younger than age 50, but not among participants aged 50 and older (Supplementary Fig. A4). Results were generally similar for males and females. Women but not men appeared to have higher PRs of depression with increasing SRAD 14 (Supplementary Fig. A5) whereas SRAD14 and SRAD30 were suggestively associated with decreasing PRs of distress among males but not females.

## Discussion

This study found that the association between mental health and solar radiation varied somewhat by the type of psychological outcome. Psychological distress may represent temporary symptoms that could be improved over a relatively short time span [26]. So, it may be more subject to short-term climate changes. On the other hand, the symptoms of depression can last for years without improvement and symptoms might be less affected by short-term changes in the level of solar radiation [26, 27]. In clinical settings, this duration difference helps distinguish depression from distress because patients with distress are more likely to make rapid improvements emotionally while depressed patients require longer-term treatment [27]. This difference may also explain why distress was more sensitive to recent solar radiation levels in this study. A longer exposure window may have captured associations between solar radiation and depression that we missed by focusing on measures of 30 days or less.

Higher levels of SRAD were suggestively associated with decreasing frequency of distress in our study. There are no prior studies evaluating the effects of solar radiation specifically on psychological distress. Most studies focused on general mood disorders. Some found beneficial effects of sunlight, and some did not [10–15]. These inconsistencies might be due to the definition of outcomes such as the failure of differentiating psychological distress from other mood disorders [28]. In addition, climate adaptation in different study areas and differences in the demographic composition may also contribute to differences across studies that had different ages, races, education, income, and levels of background solar radiation. Furthermore, exposure misclassification from using regular monitoring stations could impact the validity of prior findings. Besides, we found a potential U shape effect for SRAD7 where we observed more apparent beneficial effects for Q2 and Q3 but not Q4 associated with both outcomes. Similarly, Kent et al. [11] identified a U-shaped effect of solar radiation on depression [11]. In their study, the OR of the lowest radiation (<10,000 J/m^2^) = 1.14, and the ORs of the radiation (10,000-25,000 J/m^2^) = 0.87–0.94 with the highest radiation as the reference (20,000-25,000 J/m^2^, OR = 1). This pattern could reflect behavioral differences, such as staying indoors when it is scorching hot.

The beneficial association between solar radiation and mood disorders found in this study could be due to modulation of neurotransmitters. Evidence has shown that mood disorders are closely related to dopamine homeostasis, and ultraviolet radiation could accelerate dopamine release [29–31]. Sunlight is related to vitamin D, and other evidence suggests that as an antioxidant in brain tissue, decreased Vitamin D levels are related to increased symptoms of depression and anxiety [32]. Akpınar et al. [32] suggested Vitamin D screening for prevention and treatment of mood disorders [32]. Mood disorders have been consistently linked to disrupted circadian rhythm via multiple pathways such as monoamine signaling, immune function, HPA axis regulation, and metabolic peptides [33].

We found suggestive evidence that the effects of solar radiation on depression and distress vary by season. Exacerbated risk effects were found in the Spring and Summer while beneficial effects were observed in the Fall (Sept. to Nov.). Similarly, some studies suggested that mood disorders such as bipolar disorders are seasonal and can be affected by the change in solar radiation [34]. In the Spring and Summer, as the summer solstice approaches and the longest days of the year occur, increased energy levels followed by changes in mood could lead to symptoms of hypomania or mania [34]. These non-specific symptoms may be similar to components of psychological distress such as stress and anxiety. In contrast, Fall is the transitional season with temperature and energy starting to decrease, which may lead to a depressed mood. In this case, a higher level of solar radiation could help these depressive symptoms showing beneficial effects [34]. Rosenthal et al. [35], reported that treatment during the transition from winter to spring (solar radiation and temperature increase over time) could differ from strategies during the transition from summer to fall (solar radiation and temperature decrease over time) [35]. These studies provide support for seasonal variations in the association between solar radiation and mood disorders. In addition, compared to females, males were more likely to benefit from high solar radiation, which may relate to the enhancement of hormones by sun exposure and active outdoor activities [36, 37]. Parikh et al. [36] found that sun exposure could affect hormone release in males but not in females [36]. Upon sun exposure, there is an enhancement of lipid and steroid metabolism and metabolism-associated peptides in males [36].

We found that higher level of SRAD14 is related to increasing PR of depression among females. Unfortunately, we could not find any similar results from other studies. However, previous findings have shown that females are more vulnerable to high temperature and heat waves compared to males [10]. Females have a higher core temperature after ovulation and lower capacity to sweat [38]. These features increase the body’s burden on the cardiovascular system. In addition, many of the men in our study worked as fishermen or in other trades that required them to work outdoors. The same is not true for females in the study sample who may have been more likely to stay indoors in periods of high solar radiation where they would not experience the beneficial effects of exposure to solar radiation.

To our knowledge, this is one of the few studies assessing solar radiation in relation to both psychological distress and depression. We had a relatively large sample size compared to prior similar studies [16, 39]. A larger sample size additionally allowed us to explore differences in associations by season, age, and sex. Instead of using solar radiation obtained from regular monitoring stations, we used a finer scale of solar radiation at 1 km x 1 km grid, which greatly improved the spatial resolution of exposure thereby reducing misclassification attributed to less-resolved estimates.

Several limitations should also be acknowledged. Firstly, this study is not a random or representative sample of the US Gulf region. However, we had a relatively large sample size and good spatial coverage across the region. In addition, this is a cross-sectional study. Even though we calculated solar radiation exposures for time periods prior to the assessment of mental status, the temporal link between the outcome and the exposure cannot be established, especially because the symptom period covered the prior two weeks. Moreover, exposure misclassification could still exist in this study because solar radiation exposures were obtained from modeling and were characterized at participants’ residences. This is unlikely to fully represent their true exposure given that behavior patterns and indoor activities are unaccounted for in this analysis (e.g., where they work and how much time they spend outside). However, this study represents an improvement over previous studies using limited monitoring sites. Our study used questionnaires rather than clinical assessments to characterize mental health outcomes. However, the PHQ-9 and K6 have been widely validated and used in many studies. For instance, validation studies of the K6 against clinical structured diagnosis reported an accuracy of 0.92 [20, 21]. and PHQ-9 score ≥10 yielded a sensitivity of 88% and a specificity of 88% for major depression [19].

## Conclusion

In this study, we found that higher levels of SRAD7 were non-monotonically inversely associated with depression, and increasing levels of SRAD7, SRAD14 and SRAD30 were suggestively associated with decreasing frequency of distress. The effects of solar radiation on depression and distress varied by season and associations with distress were somewhat more apparent among men. Overall, our results support a potential beneficial effect of solar radiation that should be confirmed in studies with greater geographic variation and medically confirmed diagnoses.

## Supplementary information

Appendix

## Data Availability

The datasets generated during and/or analysed during the current study are available from the corresponding author on reasonable request.
