# Supplementary material for: Association between solar radiation and mood disorders among Gulf Coast residents
Source: J Expo Sci Environ Epidemiol. 2024 Jun 3;35(5):813–20. doi: 10.1038/s41370-024-00691-w (PMC12400302; doi:10.1038/s41370-024-00691-w)
Supplement: Supplementary file 1 — Appendix [file 41370_2024_691_MOESM1_ESM.docx]

Appendix


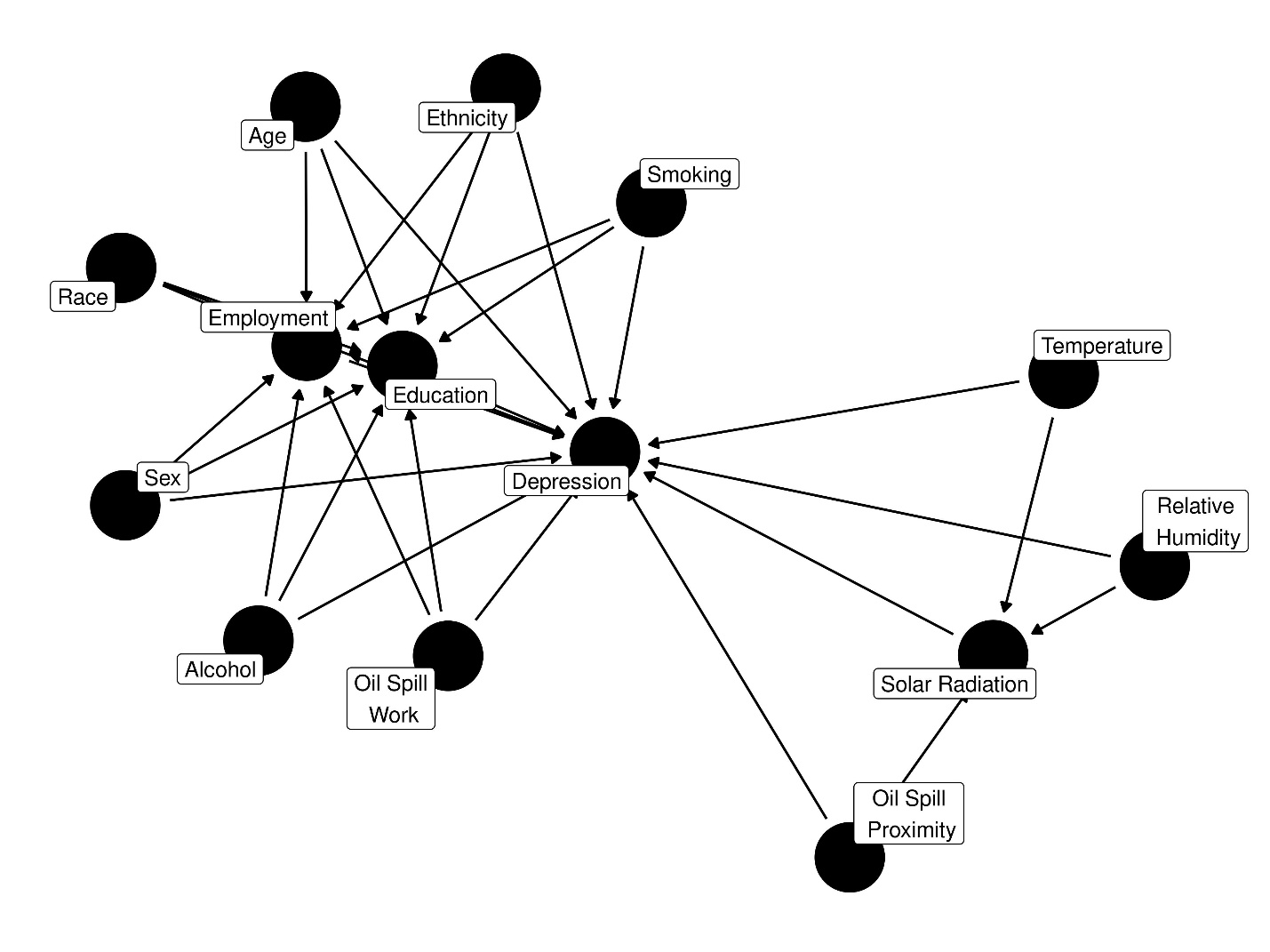


Figure A1. Directed acyclic graph.


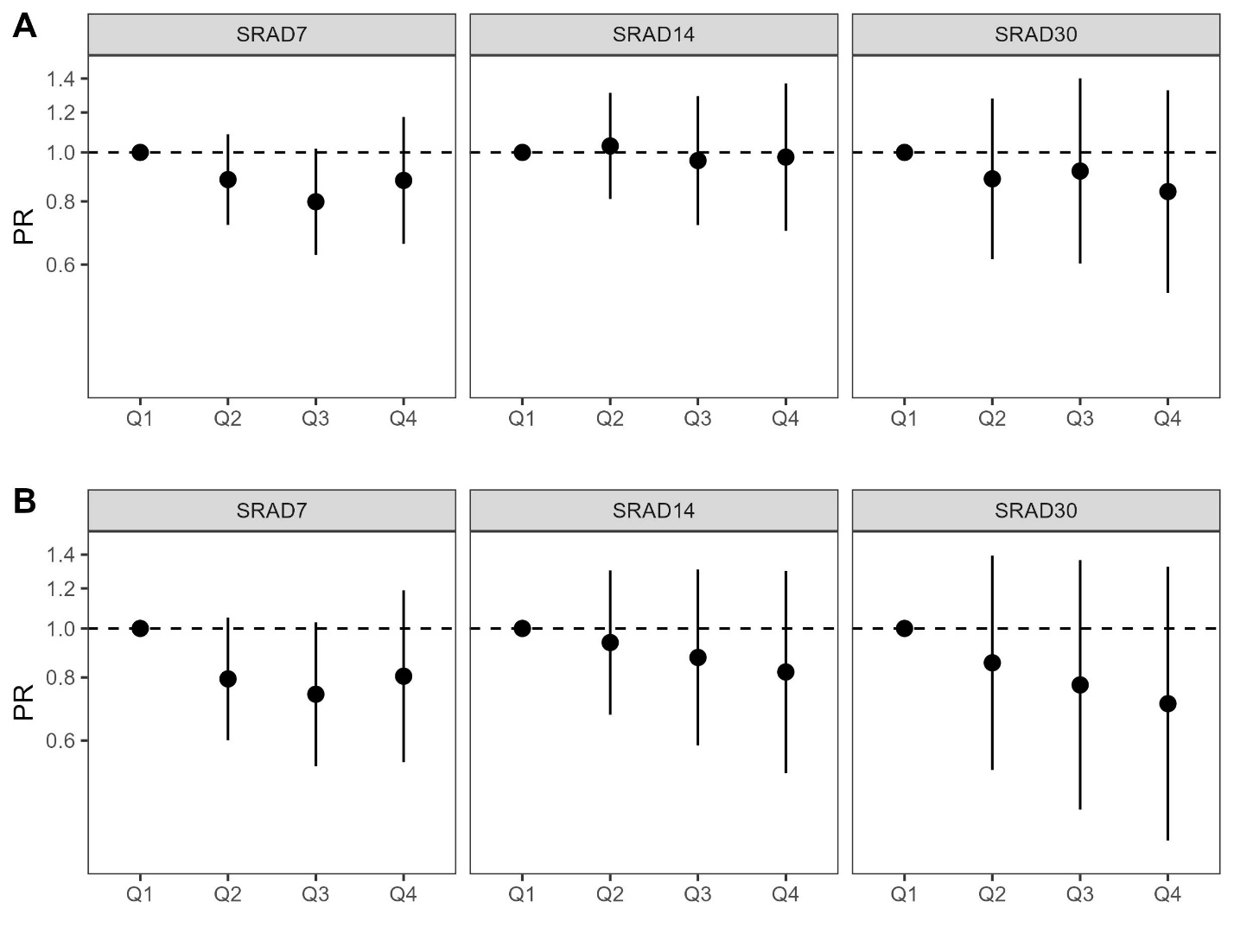


Figure A2. Association of depression and distress with quartiles of average solar radiation in the past seven, 14 and 30 days among GuLF study participants with data for both depression and distress (N = 8,446). Panel A: depression; Panel B: distress.


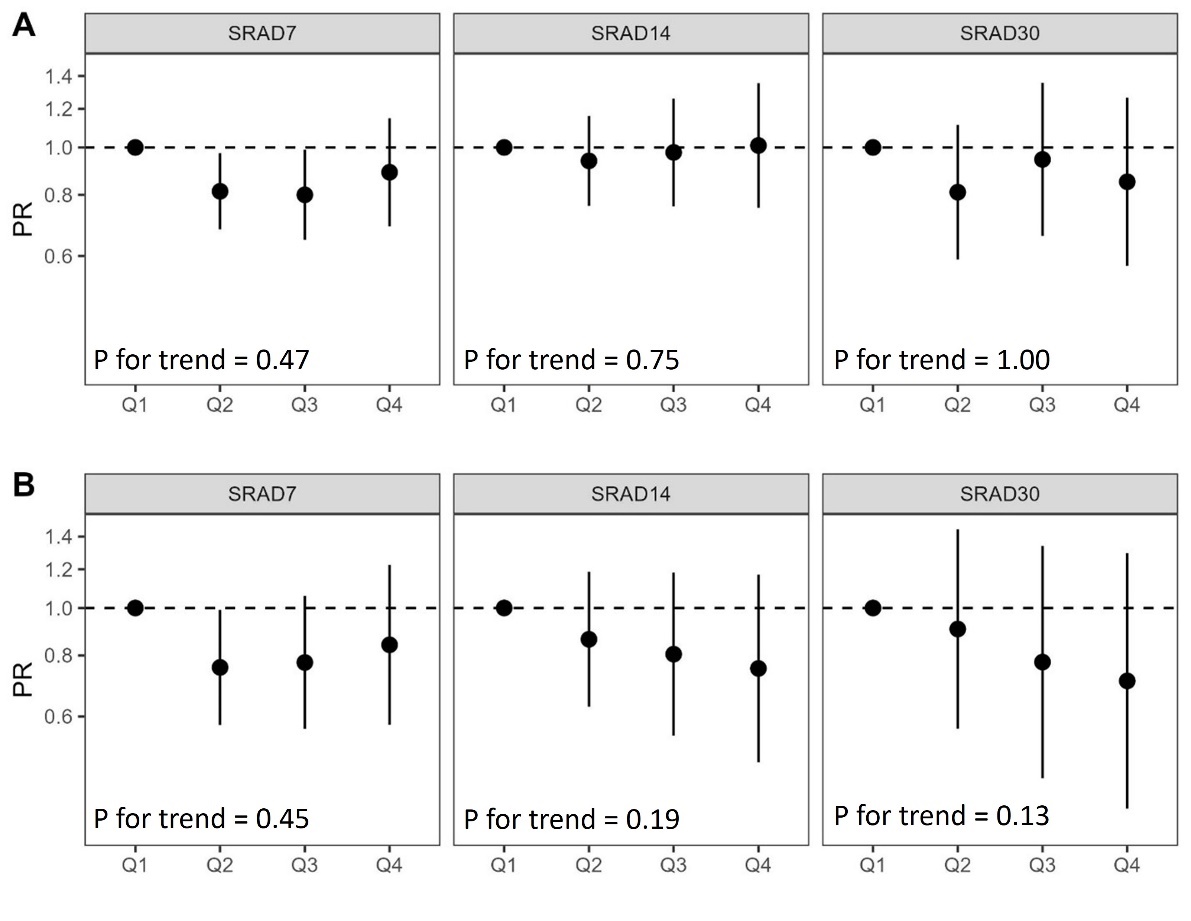


Figure A3. Association of depression and distress with quartiles of average solar radiation in the past seven, 14 and 30 days among GuLF study participants. Panel A: depression (N = 10,217); Panel B: distress (N = 8,765).


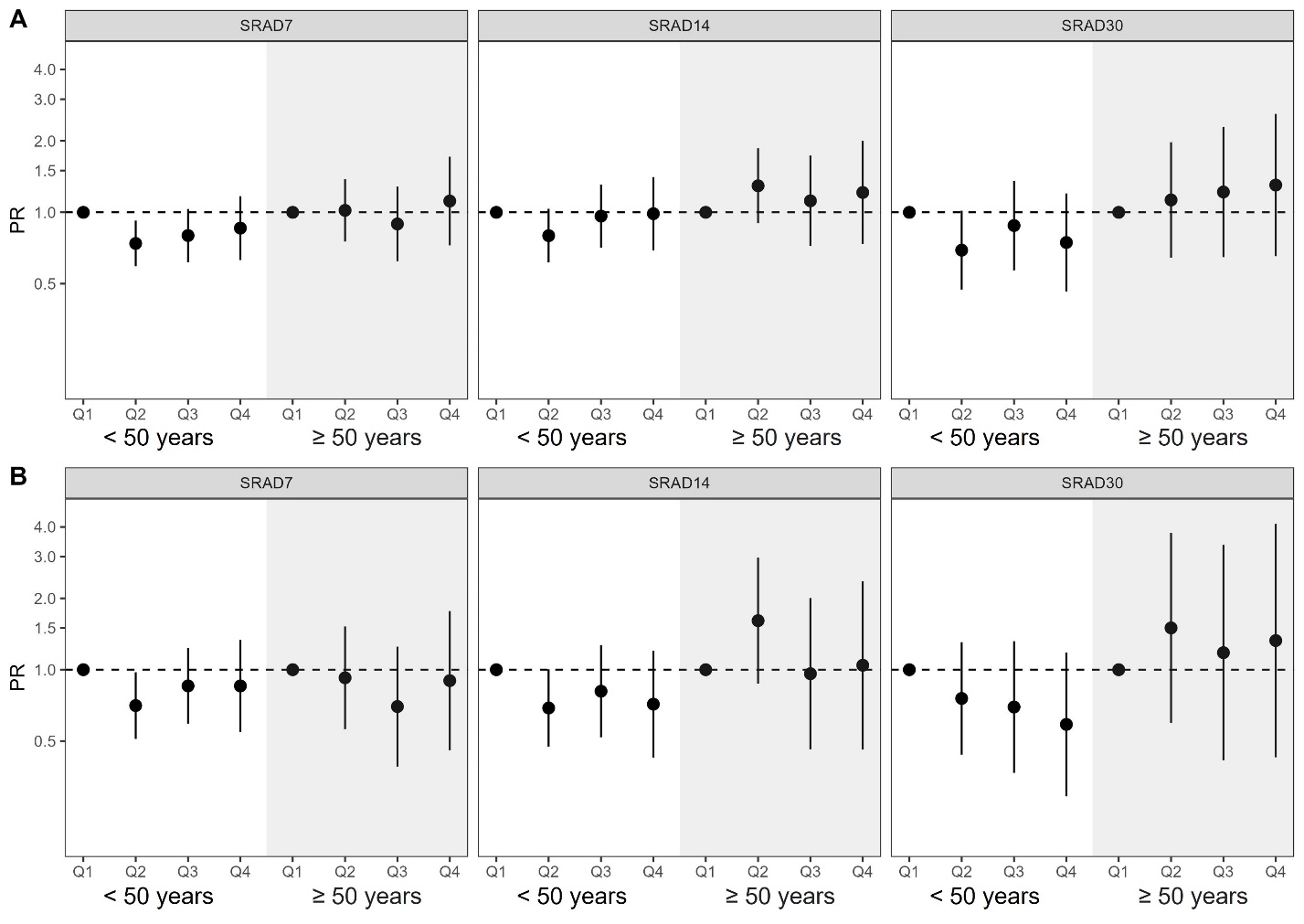


Figure A4. Association of depression and distress with quartiles of average solar radiation in the past seven, 14 and 30 days among GuLF study participants stratified by age group (<50 years; ≥50 years). Panel A: depression (N = 10,217, <50 years = 6,558, ≥50 years = 3,659); Panel B: distress (N = 8,765, <50 years = 5,648, ≥50 years = 3,117).


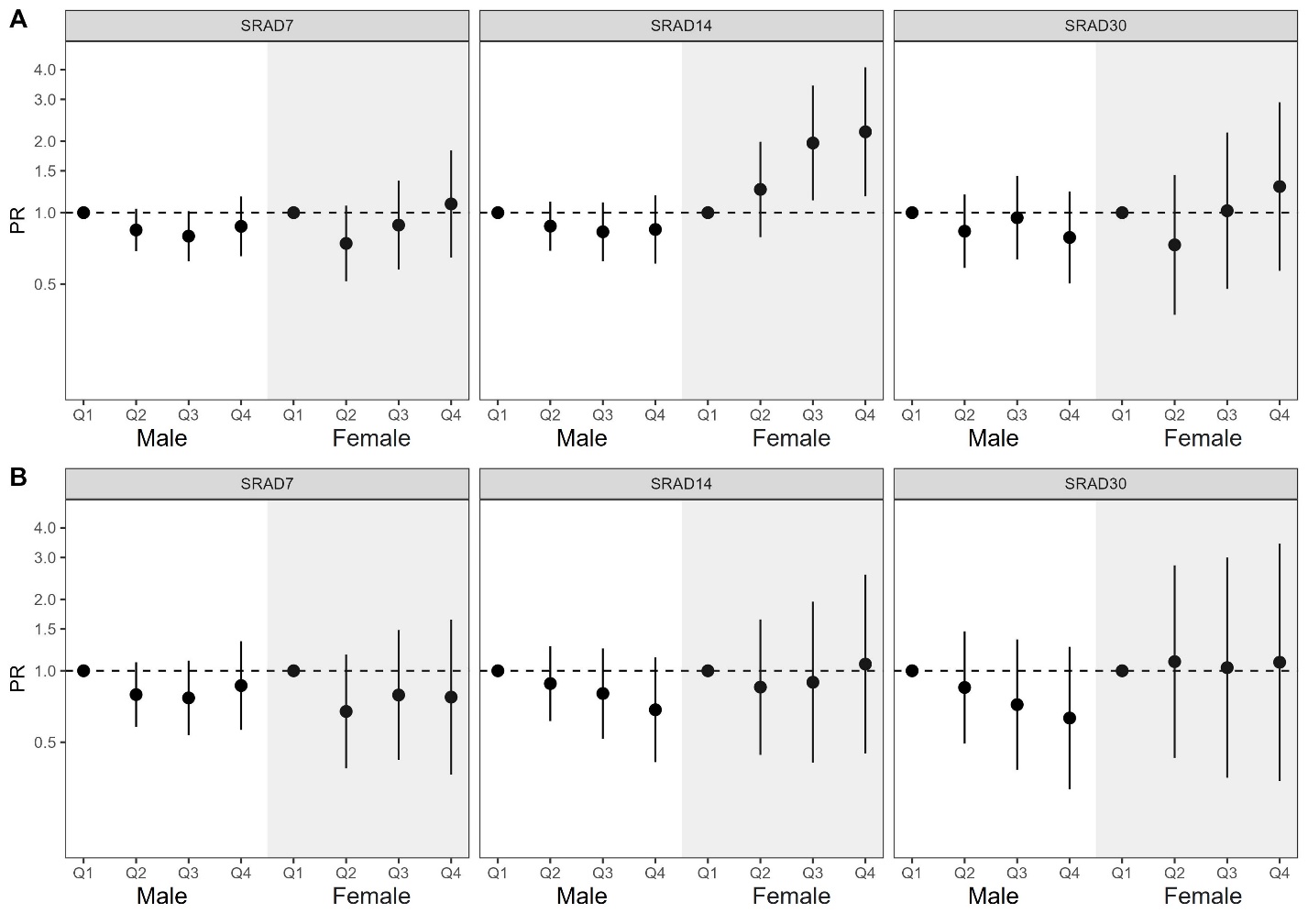


Figure A5. Association of depression and distress with quartiles of average solar radiation in the past seven, 14 and 30 days among GuLF study participants stratified by sex. Panel A: depression (N = 10,217, Male = 7,985, Female = 2,232); Panel B: distress (N = 8,765, Male = 6,828, Female = 1,937).

Table A1. Associations between solar radiation measures and mental health outcomes, with and without adjustment for income among participants with non-missing income data.

| **outcome** | | | **Crude model** |  | **Crude model + Income** |
| --- | --- | --- | --- | --- | --- |
|  |  |  | PR (95%CI) |  | PR (95%CI) |
| **PHQ9**  **(N = 9,707)** | SRAD7 | Q2 | 0.87 (0.74-1.01) |  | 0.89 (0.76-1.04) |
|  |  | Q3 | 0.89 (0.76-1.04) |  | 0.92 (0.79-1.08) |
|  |  | Q4 | 1.02 (0.88-1.19) |  | 1.04 (0.89-1.21) |
|  | SRAD14 | Q2 | 0.91 (0.78-1.06) |  | 0.93 (0.80-1.09) |
|  |  | Q3 | 0.97 (0.83-1.13) |  | 1.01 (0.86-1.18) |
|  |  | Q4 | 1.05 (0.91-1.22) |  | 1.07 (0.92-1.25) |
|  | SRAD30 | Q2 | 0.85 (0.73-0.99) |  | 0.88 (0.75-1.03) |
|  |  | Q3 | 1.10 (0.95-1.28) |  | 1.12 (0.96-1.30) |
|  |  | Q4 | 0.97 (0.84-1.13) |  | 0.99 (0.85-1.16) |
|  |  |  |  |  |  |
| **KESSLER**  **(N = 8,345)** | SRAD7 | Q2 | 0.80 (0.64-1.01) |  | 0.82 (0.65-1.03) |
|  |  | Q3 | 0.81 (0.65-1.02) |  | 0.85 (0.67-1.07) |
|  |  | Q4 | 0.93 (0.75-1.17) |  | 0.95 (0.76-1.18) |
|  | SRAD14 | Q2 | 0.88 (0.70-1.10) |  | 0.90 (0.72-1.14) |
|  |  | Q3 | 0.88 (0.70-1.11) |  | 0.92 (0.73-1.17) |
|  |  | Q4 | 0.94 (0.75-1.18) |  | 0.96 (0.76-1.20) |
|  | SRAD30 | Q2 | 0.98 (0.78-1.23) |  | 1.02 (0.82-1.29) |
|  |  | Q3 | 0.90 (0.71-1.14) |  | 0.92 (0.72-1.16) |
|  |  | Q4 | 0.94 (0.75-1.18) |  | 0.97 (0.77-1.22) |

Table A2. Associations of depression and distress with quartiles of average solar radiation in the past seven, 14 and 30 days among GuLF study participants.

| Outcome | Exposure | Quartiles | | Crude  PR (95% CI) | | Model 2*  PR (95% CI) | | Model 3^#^  PR (95% CI) |
| --- | --- | --- | --- | --- | --- | --- | --- | --- |
| PHQ9  (N = 10,217) | SRAD7 | Q2 | 0.86 (0.74-1.00) | | 0.85 (0.72-1.01) | | 0.81 (0.68-0.97) | |
|  |  | Q3 | 0.86 (0.74-1.00) | | 0.87 (0.72-1.05) | | 0.80 (0.65-0.99) | |
|  |  | Q4 | 0.99 (0.86-1.15) | | 1.02 (0.83-1.24) | | 0.89 (0.69-1.15) | |
|  | SRAD14 | Q2 | 0.90 (0.77-1.05) | | 0.97 (0.79-1.19) | | 0.94 (0.76-1.16) | |
|  |  | Q3 | 0.97 (0.84-1.13) | | 1.05 (0.84-1.32) | | 0.98 (0.77-1.26) | |
|  |  | Q4 | 1.04 (0.90-1.21) | | 1.14 (0.90-1.45) | | 1.01 (0.75-1.35) | |
|  | SRAD30 | Q2 | 0.85 (0.73-0.99) | | 0.85 (0.63-1.16) | | 0.81 (0.59-1.11) | |
|  |  | Q3 | 1.07 (0.92-1.24) | | 1.06 (0.76-1.46) | | 0.95 (0.66-1.35) | |
|  |  | Q4 | 0.98 (0.85-1.14) | | 0.98 (0.70-1.36) | | 0.85 (0.57-1.26) | |
|  |  |  | |  | |  | |  |
| Kessler  (N = 8,765) | SRAD7 | Q2 | | 0.77 (0.62-0.96) | | 0.74 (0.57-0.97) | | 0.76 (0.58-0.99) |
|  |  | Q3 | | 0.79 (0.63-0.99) | | 0.76 (0.58-1.01) | | 0.77 (0.57-1.06) |
|  |  | Q4 | | 0.89 (0.72-1.11) | | 0.82 (0.61-1.11) | | 0.84 (0.58-1.22) |
|  | SRAD14 | Q2 | | 0.89 (0.71-1.11) | | 0.89 (0.65-1.21) | | 0.86 (0.63-1.19) |
|  |  | Q3 | | 0.88 (0.70-1.10) | | 0.86 (0.61-1.22) | | 0.80 (0.55-1.18) |
|  |  | Q4 | | 0.92 (0.74-1.15) | | 0.81 (0.56-1.17) | | 0.75 (0.48-1.17) |
|  | SRAD30 | Q2 | | 0.96 (0.77-1.20) | | 0.92 (0.59-1.46) | | 0.91 (0.57-1.45) |
|  |  | Q3 | | 0.89 (0.71-1.11) | | 0.85 (0.52-1.39) | | 0.78 (0.45-1.34) |
|  |  | Q4 | | 0.91 (0.72-1.14) | | 0.79 (0.48-1.30) | | 0.71 (0.39-1.29) |

* Model 2: adjusted for age groups, race, ethnicity, gender, education, employment, alcohol consumption, smoking, oil spill worker status, season, and proximity to coast.

^#^ Model 3: adjusted for temperature, relative humidity, age, race, ethnicity, gender, education, employment, alcohol consumption, smoking, oil spill worker status, season, and proximity to coast.
